# The Surgical Dental Anxiety Scale (SDAS)

**DOI:** 10.1038/s41415-024-7846-1

**Published:** 2024-12-20

**Authors:** Sanford Grossman

**Affiliations:** Bristol Dental School, University of Bristol, United Kingdom

## Abstract

Dental anxiety is a prevalent issue in society and national surveys show it to be rising. As a result, strain on sedation services continues to grow. To accommodate this, there is a need to streamline services to ensure that patients who have a clinical need for sedation are able to receive it.

The Index of Sedation Need (IOSN) has been developed as a means of distinguishing sedation need from demand, enabling appropriate assessment and selection. NHS England recommends its use and the Getting It Right First Time programme has incorporated it into hospital dentistry pathways for sedation. However, many patients have sedation or require it for dental extractions, yet the Modified Dental Anxiety Scale (MDAS) component of the IOSN does not take this into account. While the MDAS is an effective tool for assessing anxiety of general dental treatment, it is not specialty- or procedure-specific and may underestimate anxiety relating to dental extractions. Consequently, the suitability of its use has been called into question.

This paper explores the need for a procedure-specific dental anxiety scale for use in oral surgery and outlines a proposal for a suitable model: the Surgical Dental Anxiety Scale (SDAS).

## Key points

- Discusses the prevalence of dental anxiety and its implications on sedation provision.
- Suggests that the Modified Dental Anxiety Scale may have limitations when used for sedation selection in patients undergoing dental extractions.
- Proposes a procedure-specific dental anxiety scale for use in oral surgery.

## Introduction

Dental anxiety remains a consistent barrier to dental care for a large portion of the public. The 2021 Adult Oral Health Survey found two-fifths of adults with natural teeth (42%) experienced moderate dental anxiety and a further 12% suffered from extreme dental anxiety.^1^ In comparison, the 2009 Adult Dental Health Survey reported fewer patients (36%) experienced moderate levels of dental anxiety, while the figure for extreme anxiety was unchanged (12%).^2^

In 2012, it was estimated that 5% of dental attenders will require treatment under sedation at some point in time.^3^ With the rise in moderate dental anxiety, it can be anticipated that demand for sedation and consequent strain on services will grow.

## The Indicator of Sedation Need

The Indicator of Sedation Need (IOSN) is a tool which was developed by Coulthard *et al*. in 2011, with a view to distinguish sedation need from demand. It consists of three components:^4^

- The Modified Dental Anxiety Scale (MDAS)
- Medical and behavioural indicators
- Dental treatment complexity.

NHS England is concerned that some sedation provision is being led by patient demand rather than clinical need. It therefore recommends the use of the IOSN to aid clinical assessment and treatment planning with the focus on clinical need.^5^^,^^6^

Its use is also recommended by Getting It Right First Time - a national programme designed to improve the quality of care within the NHS by reducing unwarranted variations in service. Its recommendation is that all secondary care services providing dentistry should aim to have access to a conscious sedation service in order to reduce reliance on general anaesthetic and improve patient care and choice, and that referral pathways should be set up accordingly with use of the IOSN.^7^

Despite the recommended use of the tool on a national level, several papers have questioned its validity in defining sedation need for patients undergoing dental extractions.

A study by Gerrard (2016) at a dental teaching hospital investigated patients attending for oral surgery procedures with sedation which had been treatment planned without reference to the IOSN. The relevant questionnaire was later completed by the patients and it was found that only 56% were receiving sedation appropriately in line with the tool. Additionally, 50% of patients who had no need for sedation according to the IOSN were considered by the operator to have been untreatable without it.^5^

Another study by Goodwin *et al*. (2012) deemed that 45% of patient cases where the IOSN suggested sedation wasn't needed couldn't have been completed without it.^8^

The arguments provided from these papers largely focus on the tool's use of the MDAS.

## The Modified Dental Anxiety Scale

The MDAS was first described by Humphris in 1995 (Table 1).^9^ The brief, five-item questionnaire is the most frequently used dental anxiety scale in the UK.^10^ Its validity and reliability have been tested extensively.^9^^,^^11^

**Table 1:** The MDAS. Each item scores are as follows: not anxious = 1; slightly anxious = 2; fairly anxious = 3; very anxious = 4; extremely anxious = 5. Total score is a sum of all five items, range 5-25^9^

If you went to your dentist for treatment tomorrow, how would you feel?				
Not anxious	Slightly anxious	Fairly anxious	Very anxious	Extremely anxious
If you were sitting in the waiting room (waiting for treatment), how would you feel?				
Not anxious	Slightly anxious	Fairly anxious	Very anxious	Extremely anxious
If you were about to have a tooth drilled, how would you feel?				
Not anxious	Slightly anxious	Fairly anxious	Very anxious	Extremely anxious
If you were about to have your teeth scaled and polished, how would you feel?				
Not anxious	Slightly anxious	Fairly anxious	Very anxious	Extremely anxious
If you were about to have a local anaesthetic injection in your gum, above an upper back tooth, how would you feel?				
Not anxious	Slightly anxious	Fairly anxious	Very anxious	Extremely anxious

It should be noted that not all procedures lead to the same amounts of dental anxiety. Before the MDAS, the Corah Dental Anxiety Scale (CDAS) was widely used.^12^ The CDAS did not include any reference to local anaesthetic injections - a major focus of anxiety for many. The tool could therefore overlook patients who were anxious about injections only. Humphris overcame this through development of the MDAS, including a question on anxiety about oral injections.^9^

In much the same way that the CDAS lacked reference to injections - a major source of dental anxiety - the MDAS does not reference dental extractions, which have been shown to be the greatest source of anxiety compared to other dental treatments.^5^^,^^13^^,^^14^^,^^15^^,^^16^

As a component within the IOSN, the MDAS may therefore underestimate the needs of patients who are able to tolerate general dental treatment but suffer from anxiety specifically related to dental extractions. This may lead to patients being identified as having no clinical need for sedation in line with their IOSN score, when in reality, it may be the most appropriate modality for them.

Similarly to the MDAS addressing the lack of injection-related anxiety in the CDAS, this paper proposes an oral surgery-specific variant to be used for patients needing dental extractions: the Surgical Dental Anxiety Scale (SDAS).

## Dental extraction-specific dental anxiety scales

Few attempts have been made to produce oral surgery- or dental extraction-specific dental anxiety scales. Where models have been suggested, questions have lacked specific procedural details, such as experiencing bone removal during a surgical extraction,^17^ or relevance to procedural anxiety and brevity.^18^ In any case, none have gained traction or adopted use within the UK.

## The proposed Surgical Dental Anxiety Scale (SDAS)

The proposed oral surgery-specific scale - the SDAS - is presented in Table 2.

**Table 2:** The Surgical Dental Anxiety Scale (SDAS)

If you went to your dentist for a dental extraction tomorrow, how would you feel?				
Not anxious	Slightly anxious	Fairly anxious	Very anxious	Extremely anxious
If you were sitting in the waiting room (waiting for a dental extraction), how would you feel?				
Not anxious	Slightly anxious	Fairly anxious	Very anxious	Extremely anxious
If you were about to have a local anaesthetic injection in your gum, how would you feel?				
Not anxious	Slightly anxious	Fairly anxious	Very anxious	Extremely anxious
If you were about to have a ‘simple' tooth extraction (using forceps), how would you feel?				
Not anxious	Slightly anxious	Fairly anxious	Very anxious	Extremely anxious
If you were about to have a ‘surgical' tooth extraction (raising a gum flap, removing bone with a drill, stitching), how would you feel?				
Not anxious	Slightly anxious	Fairly anxious	Very anxious	Extremely anxious

While the MDAS gives an overall score to assess anxiety about general dentistry with only some insights into anxiety relating to particular procedures, the SDAS is oral surgery-specific and largely investigates a patient's feelings around dental extractions.

Attempts have been made to keep the structure and format of the SDAS closely related to that of the extensively tested MDAS, with changes only where necessary to produce a procedure-specific variant.

In the MDAS, questions are presented in order of increasing anxiety, culminating with feelings around what was perceived to be the greatest source of anxiety: a local anaesthetic injection in the gum. In keeping with this, the order of increasing anxiety is maintained in the SDAS, naturally shifting the local anaesthetic question up in place of questions pertaining to simple and surgical extractions, respectively.

Key descriptors have been added with terminology often used with patients (eg ‘raising a gum flap', ‘removing bone with a drill') in order to gauge their anxiety towards varying complexity dental extractions (ie simple or surgical). This language is provided in a context they are likely to understand and that will hopefully have been discussed with them at the time of their consultation. By referencing the raising of a flap, use of a handpiece and suturing, most aspects of a surgical extraction from a patient's point of view are included to assess anxiety levels for a more complex extraction procedure.

The validity of certain components of the MDAS could be directly applied to unchanged elements of the SDAS, for instance, the sequence of answers and use of a Likert scale.^9^ However, research is warranted to establish the reliability and validity of the SDAS and determine an overall score reproducibly consistent with sedation need.

In some cases, the SDAS will clearly highlight a need for sedation without use of a calibrated score. For example, if a patient were to tick ‘extremely anxious' to question five (‘if you were about to have a “surgical” tooth extraction [raising a gum flap, removing bone with a drill, stitching], how would you feel?'), it could be assumed that they will benefit from sedation for a surgical extraction. However, the value from using such a scale with a calibrated sedation score will be in determining if on balance sedation is deemed a suitable choice in managing moderate levels of dental anxiety in patients with extraction needs. This of course will need consideration alongside the other components of the IOSN (medical and treatment complexities).

Even with extensive reliability and validity testing, it is unreasonable to expect a scale to entirely replace clinical acumen. There will be cases where sedation is considered to be appropriate despite low scores, or where the questionnaire cannot be completed, such as due to patients lacking capacity. In any case, clinical judgement, expertise and experience should remain at the forefront of the decision-making process, and tools should function as supportive measures to aid clinical assessment, rather than take full responsibility for it.

## Conclusion

The aim of this paper is to stimulate discussion around the use of the MDAS and alternative, procedure-specific scales for use in the sedation assessment process. Reliability and validity studies are needed for the SDAS and until then, its clinical application cannot be recommended. Following appropriate testing, its use could be considered for a specialty-specific dental anxiety scale to aid the decision-making process around selection for sedation.
